# Effects of Prenatal Leydig Cell Function on the Ratio of the Second to Fourth Digit Lengths in School-Aged Children

**DOI:** 10.1371/journal.pone.0120636

**Published:** 2015-03-06

**Authors:** Takahiko Mitsui, Atsuko Araki, Ayako Imai, Sakiko Sato, Chihiro Miyashita, Sachiko Ito, Seiko Sasaki, Takeya Kitta, Kimihiko Moriya, Kazutoshi Cho, Keita Morioka, Reiko Kishi, Katsuya Nonomura

**Affiliations:** 1 Department of Urology, Hokkaido University Graduate School of Medicine, Sapporo, Hokkaido, Japan; 2 Hokkaido University Center for Environmental and Health Sciences, Sapporo, Hokkaido, Japan; 3 Department of Public Health, Hokkaido University Graduate School of Medicine, Sapporo, Hokkaido, Japan; 4 Department of Obstetrics and Gynecology, Hokkaido University Graduate School of Medicine, Sapporo, Hokkaido, Japan; University of Goettingen, GERMANY

## Abstract

Prenatal sex hormones can induce abnormalities in the reproductive system and adversely impact on genital development. We investigated whether sex hormones in cord blood influenced the ratio of the second to fourth digit lengths (2D/4D) in school-aged children. Of the 514 children who participated in a prospective cohort study on birth in Sapporo between 2002 and 2005, the following sex hormone levels were measured in 294 stored cord blood samples (135 boys and 159 girls); testosterone (T), estradiol (E), progesterone, LH, FSH, inhibin B, and insulin-like factor 3 (INSL3). A total of 350 children, who were of school age and could be contacted for this survey, were then requested via mail to send black-and-white photocopies of the palms of both the left and right hands. 2D/4D was calculated in 190 children (88 boys and 102 girls) using photocopies and derived from participants with the characteristics of older mothers, a higher annual household income, higher educational level, and fewer smokers among family members. 2D/4D was significantly lower in males than in females (p<0.01). In the 294 stored cord blood samples, T, T/E, LH, FSH, Inhibin B, and INSL3 levels were significantly higher in samples collected from males than those from females. A multivariate regression model revealed that 2D/4D negatively correlated with INSL3 in males and was significantly higher in males with <0.32 ng/mL of INSL3 (p<0.01). No correlations were observed between other hormones and 2D/4D. In conclusion, 2D/4D in school-aged children, which was significantly lower in males than in females, was affected by prenatal Leydig cell function.

## Introduction

The ratio of the 2^nd^ finger to 4^th^ finger lengths (2D/4D) in humans has been reported to be smaller in males than in females [1]. This sexual difference has been attributed to the prenatal hormonal environment, such as exposure to higher levels of androgens and some other gonad-specific hormones [2] through androgen receptors, which are located in fetal cartilaginous tissue [3]. This hypothesis for the underlying mechanism for this difference is supported by the following findings; lower 2D/4D in girls with congenital adrenal hyperplasia [4], higher 2D/4D in individuals with complete androgen insensitivity syndrome [5], and the existence of a relationship between 2D/4D and polymorphisms in androgen receptors [6].

Prenatal exposure to sex hormones is known to affect human development, including that of the fetal digits, and one of the most important periods for the fetus is from the first to second trimester of pregnancy. Although most organ systems are developing during this period, the endocrine control systems have already been formed. The sexual difference in 2D/4D has already been established during early prenatal development under the influence of sex hormones [7, 8], and 2D/4D is considered to be stable after the early prenatal stages. Therefore, 2D/4D has been used as an easily measurable and stable anthropometric index of prenatal androgen exposure. However, the mechanism responsible for the sexual difference in 2D/4D has not yet been elucidated in detail.

There is currently no established approach for measuring prenatal hormone exposure when investigating the relationship between 2D/4D and the hormonal environment earlier in pregnancy in order to elucidate the mechanism underlying the sexual difference in 2D/4D; measuring prenatal hormone levels is difficult and not feasible for ethical reasons during a normal pregnancy. On the other hand, umbilical cord blood is obtained immediately after delivery, and its hormone levels are broadly considered to reflect the hormonal environment of the fetus at late gestation [9, 10]. Previous studies have been performed using cord blood to investigate the relationship between fetal hormonal exposure and human development [11–13].

In the present study, as a part of the Sapporo Cohort, Hokkaido Study on Environment and Child Health [14, 15], we investigated whether sex hormone levels in cord blood influenced 2D/4D in school-aged children.

## Participants and Methods

### Participants

This prospective birth cohort study was based on the Sapporo Cohort, Hokkaido Study on Environment and Child Health [14, 15]. Study details regarding the population, data collection, sampling of biological specimens, and contents of the questionnaire have been described previously [14, 15]. Briefly, native Japanese women living in Sapporo City or its surrounding areas were enrolled into the study at 23–35 weeks of gestation at Sapporo Toho Hospital between July 2002 and October 2005. Of the 1796 women approached, 25% were excluded as they decided to enroll in the Japanese cord blood bank or deliver the baby at another hospital; therefore, 514 pregnant women were enrolled in this cohort study (participation rate of 28.6%).

This study was approved by the Institutional Ethical Board for Epidemiological Studies at Hokkaido University Graduate School of Medicine and Hokkaido University Center for Environmental and Health Sciences. All participants provided written informed consent. Informed consent on behalf of the children enrolled was provided by their parents.

### Measurement of 2D/4D

Ten out of 514 participants were excluded from the study due to miscarriage, stillbirth, relocation, or voluntary withdrawal from the study before delivery. As 7 sets of twins were born, a total of 511 children (246 males and 265 females) were finally included in the Sapporo Cohort study. Of these, 350 children (68.1%), who are currently school-aged and could be contacted for this survey, were requested via a mail to send black-and-white photocopies of the palms of both the left and right hands. Measurements of digits were made from photocopies of the ventral surface of the right and left hands. The participants were instructed to straighten their fingers and lightly place their hands palm down on the photocopy machine. Measurements were made to the nearest 0.5 mm from the mid-point of the finger crease proximal to the palm to the tip of the finger using steel Vernier calipers. The ratio was calculated by dividing the length of the second digit by that of the fourth digit[1]. All measurements were taken twice by two observers blinded to participants’ information in order to confirm the measurements obtained.

### Sex hormone measurements in cord blood samples

At the time of delivery, a blood sample of 10–30mL was collected from the umbilical cord and stored at -80°C for later analysis.

The following hormone levels in 294 stored cord blood samples (135 boys and 159 girls) were measured. Testosterone (T), estradiol (E), and progesterone (P) levels were measured using LC-MS/MS [16, 17]. An immunoradiometric assay was used to measure luteinizing hormone (LH) (Spac-S LH Kit, TFB, Inc., Tokyo Japan) and follicle-stimulating hormone (FSH) levels (Spac-S FSH Kit, TFB, Inc., Tokyo Japan). Inhibin B levels were measured using an enzyme-linked immunosorbent assay (Inhibin B Gen II ELISA, Beckman Coulter, Inc., CA, USA). An enzyme immunoassay (Insulin-like 3 (INSL3) / RLF (Human)—EIA Kit, Phoenix Pharmaceuticals, Inc. CA, USA) was used to measure INSL3 levels. INSL3 was measured in males because it reflects Leydig cell function. It was also measured in 20 randomly selected samples from females. All sex hormone measurements were performed by Aska Pharma Medical Co., Ltd. (Kanagawa, Japan).

### Statistical analyses

Data on the characteristics of participants, 2D/4D, and sex hormone levels were presented as a group mean ± standard deviation and were analyzed between groups using a one-way ANOVA. Sex hormones were converted to a log10 scale as these data did not fall into a normal distribution. A half of the detection limit was used when levels were below the detection limit for individual hormones. The relationship between 2D/4D and sex hormone levels in cord blood samples was calculated using a multiple linear regression analysis. The inclusion of covariates was based on biological considerations and adjustments were made for maternal age (continuous), birth weight (continuous), maternal smoking during pregnancy (yes or no), and maternal alcohol consumption during pregnancy (yes or no). All statistical analyses were performed using JMP pro 10 (SAS institute Inc., NC, USA), except for the intra-class correlation coefficient for right and left 2D/4D measurements, which was calculated using SPSS statistics version 19 (IBM, IL, USA). Significance levels were set to 0.05 for all comparisons.

## Results

### 1) Patient characteristics

A total of 190 children from the 189 participants, including 88 males and 102 females, sent back photocopies of their palms. The characteristics of the participants and their children who sent back photocopies for 2D/4D were compared to their children without 2D/4D. 2D/4D was derived from the following participants; older mothers, a higher annual household income, higher educational level, and fewer smokers among family members. No significant differences were observed in gender, birth weight, or gestational age (Table 1).

**Table 1:** Patient characteristics.

		2D/4D (+)		2D/4D (-)		
		n	Mean ± SD	n	Mean ± SD	
Maternal characteristics						
Age at delivery (years old)		189	31.4 ± 4.2	315	30.7 ± 5.2	**
Pre-pregnancy BMI (m^2^/kg)		189	21.0 ± 3.1	315	21.6 ± 3.4	
Parity	Primiparous	92 (48.7)		148 (47.0)		
	Multiparous	97 (51.3)		167 (53.0)		
Annual house hold income (million yen per year)	<5	108 (57.1)		237 (75.2)		**
	≥5	81 (42.9)		78 (24.8)		
Educational level (years)	≤12	58 (30.7)		166 (52.7)		**
	≥13	131 (69.3)		149 (47.3)		
Smoking during pregnancy	Nonsmoker	174 (92.1)		232 (73.7)		**
	Smoker	12 (7.9)		83 (26.3)		
Alcohol consumption during pregnancy	Nondrinker	120 (63.5)		235 (74.6)		
	Drinker	69 (36.5)		80 (25.4)		
Infant characteristics						
Gender	Males	88 (46.3)		158 (49.2)		
	Females	102 (53.7)		163 (50.8)		
Birth weight (g)		190	3037.6 ± 379.7	321	3003.9 ± 444.5	
Gestational age (weeks)		190	38.9 ± 1.5	321	38.6 ± 1.6	

The values in brackets represent percentages.

**: p<0.01.

### 2) 2D/4D

**Fig 1:**
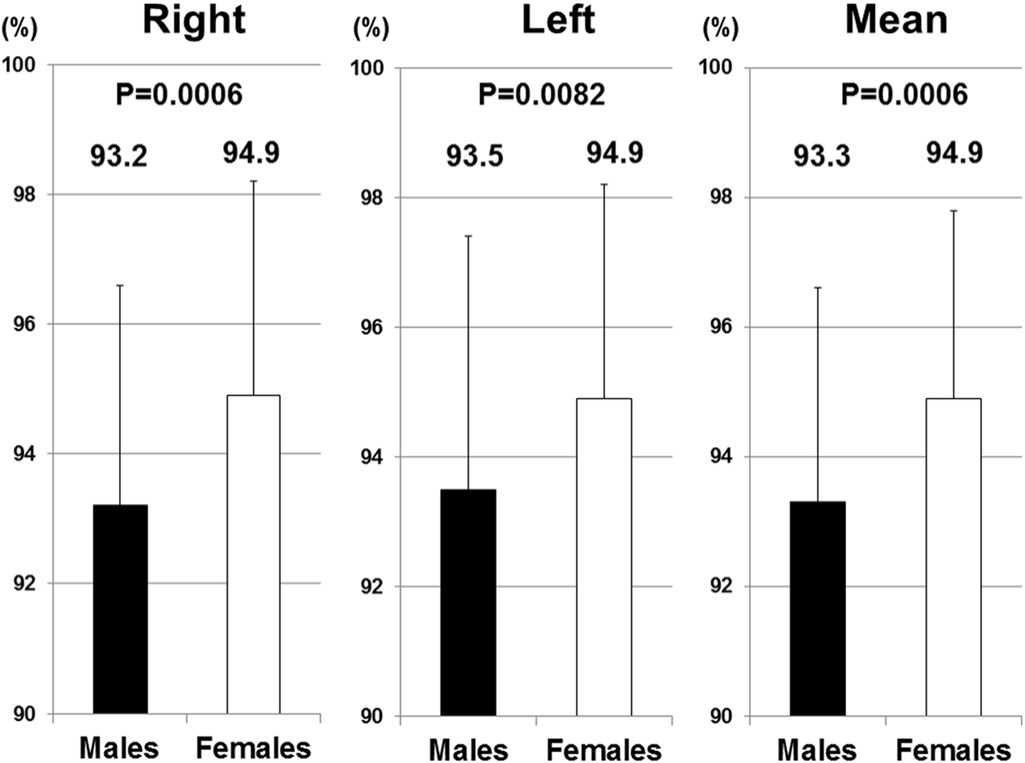
2D/4D in right hands, left hands, and mean values. 2D/4D in right hands, left hands, and mean values were significantly higher in females than in males.

In all right hand, left hand, and mean values, 2D/4D was significantly higher in females than in males (Fig. 1). 2D/4D fell into a normal distribution in all right hand, left hand, and mean values.

The intra-class correlation coefficient (1, 2) for right and left 2D/4D measurements was 0.720 (95% confidence interval: 0.627–0.789). The mean 2D/4D value in both hands was used to determine its relationship with sex hormones as a representative value of each participant.

### 3) Sex hormones in cord blood samples

T, E, P, and INSL3 were detected in all samples. INSL3 was only measured in 20 randomly selected samples from females. The detection percentages of LH in males and females were 25.7% and 0.7%, respectively, while those of FSH in males and females were 46.8% and 0%, respectively. Inhibin B was detected in 99.2% of males and 26% of females (Table 2). The mean intra-assay and inter-assay coefficients of variations in terms of sex hormone measurements were as follows; T: 1.4%–5.3%, E: 3.2%–11.3%, P: 2.7%–6.3%, LH: 4.8%–6.5%, FSH: 2.3%–3.7%, Inhibin B: < 3.8%, and INSL3: 1%–5% in the mean intra-assay coefficients of variations, and T: 3.4%–5.1%, E: 4.8%–9.5%, P: 4.7%–6.0%, LH: 7.2%–26.0%, FSH: 5.4%–6.7%, Inhibin B: < 5.6%, and INSL3: 6%–15.0% in the mean inter-assay coefficients of variations.

**Table 2:** Sex hormone levels in cord blood in males and females.

		Males				Females				
	DL	n	50^th^	25th-75th	>DL (%)	n	50^th^	25th-75th	>DL (%)	p-value
Testosterone (pg/mL)		135	98.9	76.5–126	100	156	69.9	51.9–96.3	100	<0.001
Estradiol (ng/mL)		135	4.86	3.33–7.42	100	159	4.67	3.15–6.48	100	0.227
Progesterone (ng/mL)		135	226	184–286	100	159	210	167–276	100	0.184
T/E		135	18.5	13.9–25.7	100	156	15.9	11.8–21.8	100	0.002
LH (mIU/mL)	0.5	132	<DL	<DL-0.82	25.7	155	<DL	<DL-<DL	0.7	<0.001
FSH (mIU/mL)	0.5	132	<DL	<DL-0.66	46.8	154	<DL	<DL-<DL	0.0	<0.001
Inhibin B (pg/mL)	11	134	44.0	33.9–58.3	99.2	159	<DL	<DL-11.8	26.0	<0.001
INSL3 (ng/mL)	0.01	132	0.29	0.25–0.34	100	20	0.18	0.17–0.23	100	<0.001

DL: detection limit.

The median concentrations of T, LH, FSH, Inhibin B, and INSL3, which indicate androgen activity, were significantly higher in males than in females (Table 2).

### 4) Relationship between 2D/4D and sex hormones

No significant differences were observed in the hormone levels of children who sent back photocopies for 2D/4D and those who did not (Table 3).

**Table 3:** Sex hormones in cord blood and 2D/4D.

	Males					Females				
	2D/4D (+)		2D/4D (-)			2D/4D (+)		2D/4D (-)		
	n	50^th^	n	50^th^	p-value	n	50^th^	n	50^th^	p-value
		Min		Min			Min		Min	
		Max		Max			Max		Max	
Testosterone (pg/mL)	45	90.9	90	101	0.240	69	64.9	87	71.3	0.255
		12.2		5.45			12.3		6.25	
		483		620			457		168	
Estradiol (ng/mL)	45	4.05	90	5.38	0.200	72	4.86	87	4.42	0.143
		1.91		0.01			1.66		1.44	
		26.6		33.5			31.2		17.4	
Progesterone (ng/mL)	45	183	90	234	0.378	72	201	87	216	0.457
		13.7		0.43			6.25		8.86	
		455		471			467		514	
T/E	45	21.7	90	17.5	0.477	69	15.7	87	15.7	0.424
		2.05		2.73			1.9		0.68	
		52.1		21839			47.6		40.3	
LH (mIU/mL)	45	<DL	87	<DL	0.986	70	<DL	85	<DL	0.263
		<DL		<DL			<DL		<DL	
		2.39		3.37			0.61		<DL	
FSH (mIU/mL)	45	<DL	87	<DL	0.765	72	<DL	82	<DL	N/A
		<DL		<DL			<DL		<DL	
		1.43		1.89			<DL		<DL	
Inhibin B (pg/mL)	44	43.3	90	<DL	0.957	72	<DL	87	<DL	0.947
		<DL		<DL			<DL		<DL	
		90.6		104			76.6		65.7	
INSL3 (ng/mL)	44	0.28	88	0.29	0.454		N/A		N/A	
		0.1		0.07			N/A		N/A	
		0.48		0.75						

N/A: not applicable.

**Fig 2:**
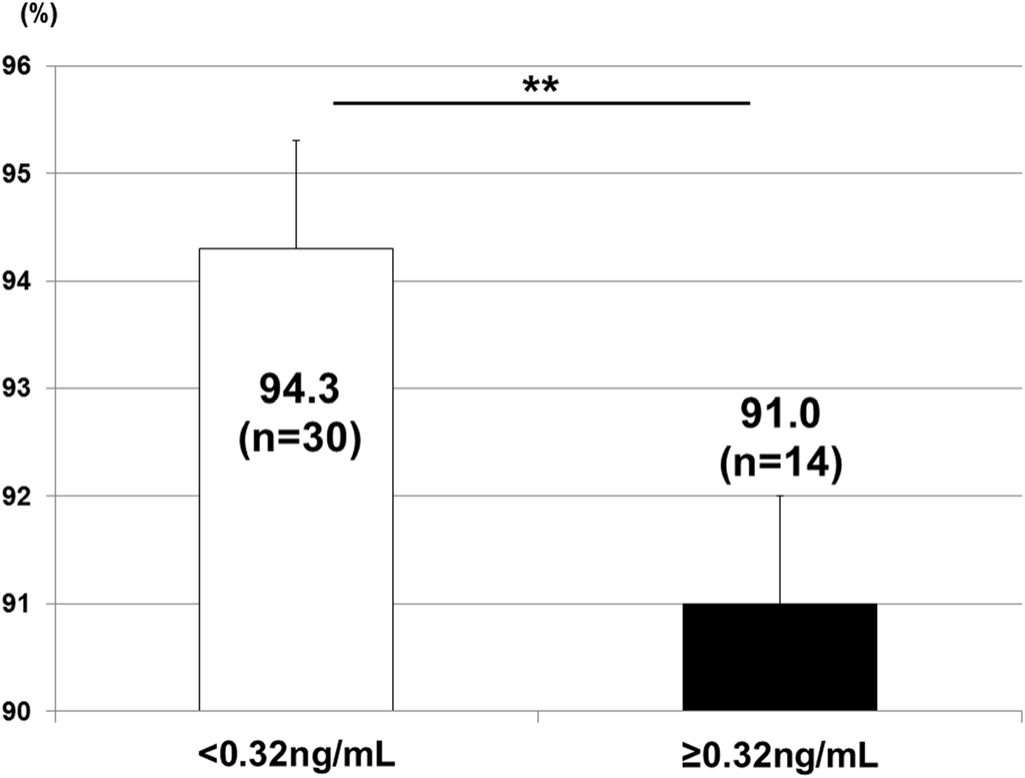
2D/4D and INSL3. 2D/4D was significantly higher in males with <0.32 ng/mL of INSL3 in cord blood (p<0.01). **: p<0.01.

A multivariate regression model showed that 2D/4D negatively correlated with INSL3 only in males. Regarding the other sex hormones in both males and females, no correlations were observed with 2D/4D (Table 4). The application of 0.32 ng/mL of INSL3 from the receiver operating characteristic curve as a cut-off value revealed that 2D/4D was significantly higher in males with <0.32 ng/mL of INSL3 (p<0.01) (Fig. 2). This result indicated that 2D/4D could be affected by prenatal Leydig cell function.

**Table 4:** Relationship between 2D/4D and sex hormones in cord blood.

Hormone levels	Total			Males			Females		
	n	B	R^2^	n	B	R^2^	n	B	R^2^
		(95%CI)			(95%CI)			(95%CI)	
**T (pg/mL)**	114	-0.021	0.113	45	-0.209	0.060	69	0.151	0.214
		(-2.449, 1.956)			(-8.080, 1.754)			(-0.835, 3.909)	
**E (ng/mL)**	117	-0.070	0.111	45	-0.051	0.022	72	-0.104	0.180
		(-2.893, 1.257)			(-4.956, 3.625)			(-3.346, 1.219)	
**P (ng/mL)**	117	0.036	0.107	45	-0.020	0.020	72	0.078	0.175
		(-1.323, 1.977)			(-4.461, 3.971)			(-1.114, 3.647)	
**T/E**	114	0.010	0.113	45	-0.138	0.036	69	0.200	0.228
		(-2.259, 2.514)			(-6.331, 2.650)			(-0.440, 5.190)	
**LH (mIU/mL)**	115	0.017	0.104	45	0.207	0.055	70	0.126	0.180
		(-2.167, 2.610)			(-1.335, 5.346)			(-6.313, 21.64)	
**FSH (mIU/mL)**	117	-0.038	0.105	45	0.180	0.048			N/A
		(-3.696, 2.448)			(-2.162, 7.177)			N/A	
**INSL3 (ng/mL)**		N/A	N/A	44	-0.377*	0.145		N/A	N/A
		N/A			(-30.17, -2.318)			N/A	
**Inhibin B (pg/mL)**	116	-0.139	0.124	44	-0.068	0.024	72	-0.082	0.172
		(-2.238, 0.331)			(-5.877, 3.891)			(-1.387, 2.732)	

*: p<0.05,

N/A: not applicable.

## Discussion

In the present study, the ratio of the digit length of the 2^nd^ finger to that of the 4^th^ finger, which has been used as an easily measurable and stable anthropometric index of prenatal exposure to androgens, was calculated in school-aged children, and sex hormone levels in cord blood samples were then measured. The levels of sex hormones indicating androgen activity in cord blood were significantly higher in males than in females. 2D/4D was significantly higher in females than in males, and negatively correlated with INSL3 only in males.

The biosynthesis of testosterone hypothetically occurs at a gestational age of 9 weeks, whereas 2D/4D dimorphism appears as early as at 14 weeks of gestation [7, 8], which indicated that early levels of sex hormones can influence 2D/4D. A previous study reported that 2D/4D reflected a genetic background subjected to a given level of exposure to prenatal androgens [1]. A gestational peak in testosterone production due to the development of Leydig cells occurred between 14 and 18 weeks. Thus, compelling evidence currently shows that 2D/4D is affected by prenatal exposure to androgens in humans.

In the present study, we used the mean 2D/4D value in both hands as a representative value of each participant, as previously reported, because the influence of the stronger side of the hands in 2D/4D on correlations with any factors has not yet been established and the intra-class correlation coefficient (1, 2) for right and left 2D/4D measurements was 0.720 (95% confidence interval: 0.627–0.789). 2D/4D in the left hand negatively correlated with INSL3 (β = -0.414, p = 0.0125), whereas 2D/4D in the right hand was not correlated with INSL3 (β = -0.268, p = 0.1093). We attributed these differences in 2D/4D between the right and left hands to various factors including measurement errors, the relatively small sample size, and the limitations associated with physical measurements. Thus, we considered it reasonable to use the mean value of 2D/4D as a representative value of each participant.

In the present study, no correlation was observed between the level of testosterone in cord blood and 2D/4D. This result was compatible with previous findings, which demonstrated that the concentration of testosterone in cord blood could not predict 2D/4D[18]. Furthermore, a previous study suggested that amniotic fluid, but not cord blood, was the best candidate for investigating the effects of early fetal exposure to androgens [19]. These findings taken together with our results indicated that testosterone in cord blood did not influence 2D:4D or reflect fetal exposure during the critical period of digit development at approximately 14 weeks of gestation. The measurement of sex hormones in cord blood may be affected by obstetric and maternal factors, such as prematurity, labor onset, placental weight, intrauterine infection, and preeclampsia, which have not yet been established in detail [9].

INSL3 levels in cord blood samples correlated with 2D/4D in males. INSL3 is constitutively produced by Leydig cells in the fetal testis, not by other organs, after sex determination [20], and is a gender-specific fetal hormone. The fetal testis is established at approximately 7 weeks of pregnancy and the *INSL3* gene in fetal Leydig cells is detectable by 8–10 weeks of pregnancy in humans [21]. This period of transition from the first to the second trimester is important for development, and is very vulnerable to a range of endocrine-disrupting insults to male reproductive development. Thus, the detection of INSL3 in fetal blood during mid-gestation reliably indicates a male fetal gender [21]. INSL3 in cord blood reflects prenatal Leydig cell function, which serves in the production of testosterone, and may also reflect androgen exposure during the important developmental window of earlier pregnancy for the digits as well as male reproductive development. In the present study, a correlation was observed between INSL3, but not testosterone, in cord blood and 2D/4D, and a previous study also demonstrated that 2D/4D was significantly related to adult testosterone levels and the presence of testosterone deficiency syndrome [22].

No correlation was noted between other hormones with androgen activity, such as LH, FSH, and Inhibin B, and 2D/4D. This may have been due to more than 50% of the stored cord blood samples being below the detection limit for LH and FSH. Therefore, more sensitive kits are needed to measure LH and FSH. Since Inhibin B reflects Sertoli cell function, its levels may not directly indicate androgen exposure *in utero* for digit development. Furthermore, a previous study using mice showed that receptors for androgen and estrogen were particularly located in the 4^th^ digit and the growth of this digit was stimulated by androgen, but arrested by estrogen[23]. Although it has already been reported that 2D/4D cannot be determined by prenatal testosterone alone and the balance between prenatal testosterone and prenatal estrogen is another important factor in fetal digit development [24], our results showed that T/E in cord blood did not correlate with 2D/4D. Thus, the present study revealed that only prenatal Leydig cell function, indicating early exposure during gestation to androgens, could be implicated in 2D/4D.

As one of factors that affects sex hormones during gestation, endocrine-disrupting chemicals, e.g. phthalates, dioxins, polychlorinated biphenyls (PCBs), and perfluorinated alkyl acids (PFAAs), have been shown to induce a broad spectrum of toxic effects on the reproductive system and genital development in the prenatal period in humans. Our cohort study already demonstrated that maternal exposure to phthalates reduced the levels of T/E, P, inhibin B, and INSL3 in cord blood, suggesting that exposure to DEHP *in utero* may have adverse effects on both Sertoli and Leydig cell development in males [25]. Previous studies also revealed that other endocrine-disrupting chemicals affected the hormonal environment during the prenatal period in humans. Cao et al. demonstrated that maternal exposure to dioxins decreased T and E in cord blood [26]. Furthermore, Hsu et al. showed that maternal exposure to PCBs decreased T/E in boys at puberty [27]. Regarding PFAAs, Vested et al. reported that maternal exposure to perfluorooctane sulfonate (PFOS) during gestation decreased the concentration and counts of sperm and increased LH and FSH levels in males after puberty, suggesting that maternal exposure to PFOS may affect semen quality and reproductive hormone levels in adult human males. Thus, maternal exposure to endocrine-disrupting chemicals influences sex hormones during gestation, as demonstrated by anti-androgen activity in males. These findings indicate that maternal exposure to endocrine-disrupting chemicals affects sex hormone levels during gestation and induces physical changes to the digits of children. An animal study has already showed that prenatal exposure to low doses of endocrine-disrupting chemicals induced feminized digit ratios in male rats[28]. Further studies are warranted to confirm this in humans.

Polymorphisms in androgen receptors (AR) may also affect sensitivity to androgen exposure in 2D/4D. AR are produced by the AR gene, which is located on the X-chromosome and repeats the nucleotide sequence CAG on exon 1. Furthermore, the number of CAG repeats varies in length among individuals and code for the length of a polyglutamine stretch on the N-terminal domain of AR. Although previous studies revealed that there was no evidence for a clear association between CAG repeats and 2D/4D [29, 30], the synergic effects of polymorphisms in AR and sex hormones in cord blood on 2D/4D remain unclear. Therefore, further investigations are needed in our cohort study.

The first limitation of this study was that we performed multiple analyses, which are associated with the risk of false positives in the main result of a correlation between 2D:4D and INSL3. The second limitation of this study was the relatively small cohort of school-aged children for whom we had data on both 2D:4D and sex hormones because only 190 (54.3%) of 350 children sent photocopies of their palms for the measurement of 2D/4D. Larger studies are needed to reveal the effects of sex hormone levels *in utero* on physical changes to children.

## Conclusions

The levels of sex hormones indicating androgen activity in cord blood were significantly higher in males than in females. 2D/4D in school-aged children, which was significantly lower in boys than in girls, was affected by prenatal Leydig cell function in males.
